# The influence of care home registration type and size on senior care leader’s confidence to provide palliative and end-of-life care: an explanatory sequential mixed methods study

**DOI:** 10.1186/s12904-024-01525-0

**Published:** 2024-08-22

**Authors:** India Tunnard, Katherine E. Sleeman, Andy Bradshaw, Anna E. Bone, Catherine J. Evans

**Affiliations:** 1 Florence Nightingale Faculty of Nursing Midwifery and Palliative Care, King’s College London, Cicely Saunders Institute, London, UK; 2 Sussex Community NHS Foundation Trust, Brighton, UK

**Keywords:** Palliative care, Nursing homes, Cross-sectional studies, Qualitative research

## Abstract

**Background:**

Care home staff are key providers of palliative and end-of-life care. Yet, little is known about how care home characteristics can influence care leader’s confidence in their ability to provide optimal palliative and end-of-life care.

**Aim:**

To understand the influence of care home registration type (nursing, residential or dual registered) and size on senior care leader’s confidence to provide palliative and end-of-life care.

**Design:**

An explanatory sequential mixed methods study comprising an online cross-sectional survey (including the Palliative Care Self-Efficacy Scale) and qualitative individual interviews. Analysis of survey data used a multivariate logistic regression and qualitative interview data used Framework Analysis. A ‘Following the Thread’ method was undertaken for data integration.

**Setting/participants:**

UK care home senior care leaders, purposively sampled by registration type, size and geographical location.

**Results:**

The online survey (*N* = 107) results indicated that nursing home senior care leaders had higher confidence scores on the Palliative Care Self-Efficacy Scale than residential care home leaders (aOR: 3.85, 95% CI 1.20-12.31, *p* = 0.02). Care home size did not show effect when adjusting for registration type (medium - aOR 1.71, 95% CI 0.59–4.97, *p* = 0.33; large – aOR 0.65, 95% CI 0.18–2.30, *p* = 0.5). Interviews (*n* = 27) identified three themes that promote confidence, (1) ‘feelings of preparedness’ stemming from staff expertise and experience and care home infrastructure, (2) ‘partnership working’ with external services as a valued member of the multidisciplinary team, and (3) a shared language developed from end-of-life care guidance.

**Conclusion:**

Care home senior care leader’s confidence is influenced by care home characteristics, particularly availability of on-site registered nurses and the infrastructure of large care homes. All care home leaders benefit from training, working with external, multidisciplinary teams and use of guidance. However, mechanisms to achieve this differed by care home type and size. Further exploration is needed on successful integration of palliative care services and interventions to enhance confidence in residential care homes.

**Supplementary Information:**

The online version contains supplementary material available at 10.1186/s12904-024-01525-0.

## Introduction

Internationally, care homes, or long-term care facilities, are an important place at the end-of-life for many older people [1]. In the UK, it is estimated that over 370,000 people live in care homes [2, 3]. Care homes provide 24-hour care to residents either with on-site registered nurses (nursing home), without registered nurses (residential home) or they are dual registered (care homes with nursing and residential beds). Over 70% of care homes in the UK are residential care homes [3, 4]. On average, UK care homes have 29.5 beds, but this can vary from less than ten to over 200 beds [5]. Care home residents are typically in the last 1–2 years of life. The life expectancy upon admission to a residential care home is 24 months, and 12 months in a nursing home [6]. On average, 22% of all deaths in the UK occur in care homes. However, during the first wave of the COVID-19 pandemic, deaths in care homes increased from 2,451 to 7,911, an increase of 220% [7]. This resulted in a rapid rise in the demand for palliative and end of life care to alleviate distressing symptoms, support recovery and anticipate and manage end-of-life care.

Recognising care homes as key providers of palliative and end-of-life care, the European Association of Palliative Care (EAPC) advocates for high-level palliative care training for all staff [8] to meet ten outlined competencies of palliative care. The aim of these competencies is *‘to see healthcare professionals grow in confidence so that they are able to anticipate palliative care needs*,* respond effectively*,* and understand their own limitations and the need to seek help’* [9]. The terms self-efficacy and confidence (in the context of ability to provide palliative and end-of-life care) are used interchangeably within this paper. Self-efficacy is the belief in one’s own capacity to engage in a certain behaviour [10]. This is influenced by confidence to attain an objective and achieve desired behavioural change [11]. The Palliative Care Self-Efficacy Scale is a measure to assess a practitioner’s self-efficacy, as influenced by confidence and belief in ability, to provide palliative and end-of-life care. The self-efficacy scale was taken from a larger series of tools for palliative care providers [12] and validated by Phillips et al. [11]. The scale incorporates aspects of the EAPC’s core competencies. It asks respondents to score their degree of self confidence in managing the physical, social and psychological needs of residents and their families, and responding to challenges of clinical decision-making.

Despite care homes being key providers of palliative and end-of-life care, there is little published evidence on care home staff confidence to deliver this care. The capability of senior care leaders in care homes to provide palliative and end-of-life care is considered a key marker of and influence on the provision of care within the care home [13, 14]. However, examination of their confidence to deliver care is often overlooked. Further, there is little consideration of how care home characteristics influence confidence. Much of the existing literature on care home staff confidence focuses on nursing homes [15–17]. However, given that the majority of UK care homes are residential, without access to on-site registered nurses [3, 4], it is vital to examine the confidence levels of residential care homes and how levels may differentiate from nursing care homes. Finally, wide heterogeneity in care home size needs to be considered as a factor influencing confidence. For example, compared with large care homes, small care homes may have limited resource and capacity to implement interventions to promote confidence, such as staff training [15].

### Aim

This study aimed to understand the influence of care home registration type and size on the confidence of senior care leaders to provide palliative and end-of-life care.

## Methods

An explanatory sequential mixed methods design, where qualitative findings are used to expand on quantitative findings [18]. Data from an online, cross-sectional quantitative survey of senior care leaders in UK care homes was used to gain a broad understanding of confidence within care homes. Subsequent individual qualitative interviews were utilised to explore and interpret quantitative findings. The Good Reporting of A Mixed Methods Study guidance [19] informed the manuscript (see additional file 1).

Bradshaw et al. [20] provide full details on the respective methods for the quantitative survey and qualitative interviews.

### Survey recruitment and data collection

Care home senior care leaders were purposively sampled. Senior care leaders were targeted to ensure the heterogeneity of the care home sector was captured and there was representation by care home registration type, size and geographical location. Individuals were invited to complete the survey via email through established care home networks, our institutional website and social media, between April-September 2021. Consent was provided by completion of the survey. The survey included open and closed-ended questions on care home characteristics, end-of-life care, support from external services and the Palliative Care Self Efficacy Scale.

#### Palliative care self efficacy scale

The Palliative Care Self Efficacy Scale [11, 21] comprises twelve items to measure confidence in providing palliative and end-of-life care. The scale is split into psychosocial (e.g. confidence in discussing wishes, answering queries and supporting the patient and family) and symptom management (e.g. ability to assess and manage symptoms) subscales (see additional file 2). The scale has an overall minimum score of 12 and a maximum score of 48. Each item has a maximum score of 4, indicating that the person is confident to perform independently, and a minimum score of 1, indicating need for further basic instruction. Previously reported Cronbach’s Alpha for the subscales were 0.87 and 0.91 respectively [11].

### Interview recruitment and data collection

All survey respondents were asked to indicate their interest in participating in a qualitative interview. Those interested were invited via email. A reminder email was sent one week later, and a telephone call the following week if no response had been received. Interviews were conducted remotely (via Microsoft Teams or by telephone) between June-October 2021 and consent given verbally and recorded. Interviews were digitally recorded and transcribed verbatim. Interviewers (IT, IB, LB) kept reflexive notes and were supervised by a senior member (CE) of the project team with qualitative expertise.

The qualitative semi-structured interview topic guide drew upon preliminary quantitative survey findings. This provided deeper exploration of areas of importance including integration with external services, working as a part of a multidisciplinary team, symptom management, family involvement and useful policies/guidance on palliative and end-of-life care.

### Data analysis

The Care Quality Commission (CQC) in England describe small care homes as comprising 1–19 beds, medium 20–49 beds and large as 50 or more beds [22]. To enable a fair distribution across groups, a data driven approach was taken to categorise this sample. Care homes were categorised as small if they had fewer than 25 beds, medium with 25–49 beds and large with 50 or more beds.

#### Quantitative data analysis

All statistical analysis was conducted in Stata SE version 17. Descriptive statistics were used to summarise demographic characteristics and calculate the median score on the Palliative Care Self-Efficacy scale. Median scores (and interquartile ranges) were used as data were not normally distributed. Total scores on the scale were dichotomised using the median score, scores ≥ 45 were considered ‘higher confidence’ and < 45 ‘lower confidence’. This method was undertaken after review of the data showed it was skewed towards the higher end of the scale. Multivariate logistic regression analyses were undertaken on total score and subscale scores.

#### Qualitative data analysis

Data from the open-ended online survey questions, interview transcripts and reflexive notes were analysed using Framework Analysis [23] to allow for comparison across care home characteristics. Analysis comprised of six steps: (1) transcription; (2) familiarisation; (3) coding; (4) developing an analytical framework; (5) application of framework; (6) charting. Researchers familiarised themselves with the transcripts, free text comments and reflexive notes (IT, ABr). An initial thematic framework was developed from preliminary analysis and agreed with the research team. Further codes were then generated inductively from the data. Transcripts were coded collaboratively, and areas of divergence discussed and agreed. Themes were charted by care home characteristics to examine variation within themes by care home size and type.

#### Integration of data

Data were integrated using a ‘following a thread’ approach [24]. Initial analysis of quantitative data identified key themes and analytical questions (the threads) for the qualitative data. Findings were pursued within and across the data sets to create a multi-faceted picture of care home staff confidence. One researcher (IT) conducted this integration, with themes agreed by the research team.

## Results

One hundred and seven care home managers and clinical leads responded to the survey. Most care homes had on-site registered nurses (54.2%; 22.4% nursing home and 31.8% dual registered). The remaining were residential only (45.8%). Survey respondents were mainly from medium (25–49 beds, 35.5%) or large (≥ 50 beds 37.4%) care homes. Similarly, the interview participants were mostly from large care homes (52.0%) and homes with on-site registered nurses (66.6%) (see Table 1). Interviews averaged 60 min (range 22–83 min).

**Table 1:** Characteristics of study participants

Characteristic		Survey respondents (*N* = 107)	Interviewees (*N* = 27)
		n (%)	n (%)
Care home size	Small (< 25 beds)	29 (27.1)	4 (14.8)
	Medium (25–49 beds)	40 (37.4)	9 (33.3)
	Large (≥ 50 beds)	38 (35.5)	14 (51.9)
Care home type	Residential	49 (45.8)	7 (25.9)
	Nursing	24 (22.4)	10 (37.0)
	Dual	34 (31.8)	10 (37.0)
Respondent role	Manager	76 (71.0)	16 (59.3)
	Deputy manager	10 (9.3)	3 (11.1)
	Registered nurse	8 (7.5)	4 (14.8)
	Other	13 (12.1)	4 (14.8)
Geographic location	East of England	7 (6.5)	-
	East Midlands	4 (3.7)	1 (3.7)
	London	35 (32.7)	5 (18.5)
	North West	8 (7.5)	3 (11.1)
	South East	20 (18.7)	8 (29.6)
	South West	8 (7.5)	2 (7.4)
	West Midlands	11 (10.3)	5 (18.5)
	Yorkshire and Humber	6 (5.6)	-
	Northern Ireland	1 (0.9)	-
	Scotland	6 (5.6)	2 (7.4)
	Wales	1 (0.9)	-

‘Other’ category included owner, area managers, directors of care, senior carers, specialist intervention workers

The Palliative Care Self Efficacy Scale median total score was 45 (IQR 39–47), indicating a generally high level of confidence in providing palliative and end-of-life care. Total and subscale scores were the lowest for small and residential care homes, indicating lower levels of confidence, and highest for medium dual registration homes (Table 2).

**Table 2:** Unadjusted analysis of palliative care self efficacy scale total and sub-scale scores by care home size and registration type

Factor		Total		Psychosocial subscale		Symptom Management subscale	
		Median score (IQR)		Median score (IQR)		Median score (IQR)	
Care home size	Small (*n = 29)*	42 (36–45)	χ^2^ (2) = 6.79, p = 0.04	22 (19–23)	χ^2^ (2) = 4.2, p = 0.12	21 (17–23)	χ^2^ (2) = 7.23, p = 0.03
	Medium (*n = 40)*	45.5 (41–48)		23 (20.5–24)		23 (20.5–24)	
	Large (*n = 38)*	44.5 (39–48)		22.5 (21–24)		22.5 (21–24)	
Care home registration type	Residential (*n = 49)*	43 (37–46)	χ^2^ (2) = 10.74, p = 0.004	21 (19–23)	χ^2^ (2) = 8.91, p = 0.01	21 (17–23)	χ^2^ (2) = 8.86, p = 0.01
	Nursing (*n = 24)*	46 (41.5–48)		23 (21.5–24)		23 (21.5–24)	
	Mixed (*n = 34)*	45.5 (41–48)		23 (22–24)		23 (22–24)	
Respondent	Manager (*n = 76)*	45 (39–47)	χ^2^ (2) = 5.53, p = 0.24	23 (20–24)	χ^2^ (2) = 2.79, p = 0.58	22 (18–24)	χ^2^ (2) = 6.00, p = 0.20
	Deputy manager (*n = 10)*	47 (44–48)		23.5 (22–24)		24 (22–24)	
	Registered nurse (*n = 8)*	45 (39-45.5)		22 (22–23)		21.5 (19-23.5)	
	Other (*n = 13)*	45 (32–48)		23 (15–24)		23 (17–24)	

‘Other’ category included an owner, area managers, directors of care, senior carers, specialist intervention workers

Unadjusted analysis signalled differences in median total scores for care home size (χ^2^ (2) = 6.79, *p* = 0.04) and registration type (χ^2^ (2) = 10.74, *p* = 0.004) (Table 2). There were differences in registration types in both subscales (χ^2^ (2) = 8.91, *p* = 0.01; χ^2^ (2) = 8.86, *p* = 0.01). However, observed differences only occurred in the symptom management scale between care homes of differing sizes (χ^2^ (2) = 7.23, *p* = 0.03).

The multivariate model showed that nursing home leaders were more confident in providing palliative and end-of-life care than residential care home leaders (aOR: 3.85, 95% CI 1.20-12.31, *p* = 0.02) (Table 3). Subscale models indicated that homes with on-site registered nurses were significantly more confident in the psychosocial subscale than residential homes (nursing *p* = 0.04; dual *p* = 0.02). Size was not shown to be a significant factor affecting confidence in any of the models.

**Table 3:** Multivariate logistic regression analysis exploring the effect of care home size and registration type on staff self-efficacy in delivering palliative and end-of-life care

Factor		Total score		Psychosocial subscale score		Symptom management subscale score	
		OR (95% CI)	*P* Value	OR (95% CI)	*P* Value	OR (95% CI)	*P* Value
Care home size							
	Small	Reference category					
	Medium	1.71 (0.59–4.97)	0.33	1.33 (0.45–3.90)	0.61	2.64 (0.90–7.73)	0.08
	Large	0.65 (0.18–2.30)	0.50	0.65 (0.18–2.35)	0.51	1.86 (0.54–6.42)	0.33
Care home registration type							
	Residential	Reference category					
	Nursing	3.85 (1.20-12.31)	0.02	3.56 (1.05–11.33)	0.04	3.06 (0.91–10.27)	0.07
	Dual	2.83 (0.90–8.89)	0.08	4.53 (1.33–15.50)	0.02	1.29 (0.42–3.91)	0.66

Multivariate logistic regression adjusted for care home size and registration type

Qualitative data provided further exploration of care home leaders confidence in providing palliative and end of life care, constructed as three main themes:

### Theme 1: ‘feelings of preparedness’ - staff expertise and care home infrastructure

Care home senior care leaders confidence in managing palliative and end-of-life care was built from feeling prepared or having an ‘*already established way of doing it’*. In large homes with on-site registered nurses, feelings of preparedness stemmed from staff experience and skills, both in duration in role and professional training, to manage care, such as administering medications. Nursing homes were able to manage symptoms and care needs within the home through established avenues of support from external palliative care services. The infrastructure in large care homes further facilitated preparedness through standardised processes, such as settling in new residents, and having appropriate equipment, such as syringe drivers, ready and available to manage needs.

*So*,* from a care home -- from an equipment point of view*,* we have got everything we really need. We’d already got [syringe] drivers*,* so we were set up for that*,* very skilled*,* confident*,* competent nurses who are very good at controlling symptoms.* [ID018 manager, large dual registration home]

Leaders in residential care homes found that the close bonds they developed with residents enabled care to be aligned with staff’s knowledge of what was important to the resident, boosting confidence in the care provided. This was complimented by training for all staff.

*I think just luckily that we had the experience of [delivering palliative and end-of-life care] in the home to begin with*,* and training and stuff we’re provided with. I think if we hadn’t have had that prior we would really have struggled. And obviously knowing the residents*,* we’re a residential home and so we’re not nursing. So knowing them as well as we did sort of helped [to deliver care].* [ID188 manager, medium residential home]

For all care home leaders, training enhanced confidence and principally came from external services, including hospice, specialist palliative care teams and specialist nurses. Training ranged from formal external courses to informal guidance on positioning or mouth swabbing delivered in the care home. The ability to access support in an ad-hoc manner was important for sustaining confidence of leaders and ensuring new staff were prepared to manage end-of-life care needs.

*we do palliative care training with the -- we have a homes’ education team with the local nurses*,* and they come in and do practical end-of-life care training. So we’ve all been trained in that*,* and our palliative care team*,* we get on so well with that*,* if I say*,* I’ve got a new member of staff*,* will you talk to them*,* they’re like*,* Yeah*,* we’ll do that with them. So we’re quite okay with that*,* and that was a bonus. They helped us perform better*. [ID231 manager, large residential home]

Interviewees advocated for the universal use of tools and guidance, such as end-of-life care plans and documentation, to be built into care home infrastructure. The introduction of the universal use of tools and guidance would encourage leaders to feel more prepared when a resident is coming to the end of their life and support equitable delivery of palliative and end-of-life care across care homes.

*So everybody should have an end-of-life care plan. Everybody should have reflected on it. It doesn’t have to be the be all and end all but it has to just be a conversation that people have so that it’s not a surprise when it suddenly happens*,* […]. But I think if people were more prepared*,* it would be so much better.* [ID018 manager, large dual registration home]

### Theme 2: ‘partnership working’ - integration with external services

For all care homes, GPs (medical doctors external to the care home) were the main external service that supported them to deliver palliative and end-of-life care. Over 76% of survey respondents sought palliative and end-of-life care advice from GPs (73.5% of residential homes, 79.2% of nursing, 79.4% of dual; 79.6% of small, 87.3% medium, 61.6% large) (see additional file 3). Nursing home interviewees indicated that their professional status as a registered nurse influenced the establishment of a trusting relationship with GPs. Regular contact with a single GP practice further strengthened trust in the nurses’ expertise and enabled staff to deliver care quickly and efficiently.

*As a nursing home we were very lucky. We have one surgery who looks after all our patients here. That means that we know the doctors very well and they know us. So they are able to trust us when we say there’s an issue. Let’s say for example somebody has a urine infection. We would ask for antibiotics and we would get it. They don’t necessarily have to come and see them.* [ID026 deputy manager, large dual registration home]

Interviewees from nursing homes further described the need for a trusting relationship with the GP to facilitate anticipatory prescribing for symptom management at the end-of-life and the revalidation of ‘just in case’ prescriptions for symptom exacerbation in chronic disease. Holding these medications within the home allowed nursing staff to feel confident that they could manage symptom distress as it arose.

*We have always*,* where we felt there was a risk that somebody is coming to the end of their life*,* requested anticipatory medications. They’ve always been given very willingly. And they have also given us a P2 [last days of life medication and administration chart] to go along with that. So any time as a nurse*,* I clinically feel it’s time to start those medications*,* we would start them. We would just let the doctor know that it was happening*,* and we would talk to the family and explain what was happening*,* and why we were giving certain medication*. [ID026 deputy manager, large dual registration home]

Residential home leaders felt valued when the GP recognised their intrinsic knowledge of residents and their role in providing palliative and end-of-life care. This included the GP consulting leaders during clinical reviews of a resident’s symptoms and concerns. Recognition of care home leader’s value was further demonstrated in leaders being invited to contribute to clinical reviews as an active member of the multidisciplinary team.

*we have weekly GP rounds where we manage symptoms; in terms of where we pick up any symptoms and report it to the GP and so we’re always a step ahead. […] we also discuss any of these symptoms in the [multi-disciplinary team] -- where we discuss any difficulties of any symptoms of which we know or anything which is untoward*,* and then how are we going to manage it. So we have a good system of support.* [ID141 manager, small residential home]

Senior care leaders reported feeling unsupported when GPs in-person visits were reduced during the pandemic, or in some cases removed completely. Without in-person support from the GP, leaders sometimes lacked confidence in their ability to make clinical judgements, with nurses occasionally requiring ‘*a second pair of eyes’*. This support was especially important in small care homes when there was only one nurse on shift.

*So really out of hours was really [only] me as a registered nurse*,* needing another informed opinion about my clinical observations.* [ID071 registered nurse, small nursing home]

In contrast, residential homes relied on GPs to manage all the healthcare needs of their residents. Where support was unavailable, this led to feelings of inadequacy, particularly in large care homes where there was a large volume of experienced carers but who were not clinically trained.

*I just didn’t feel I was adequate enough*,* I didn’t have clinical eyes* [ID111 manager, large residential home].

Good working relationships with other external services also encouraged staff to feel more confident in their role and expertise. ‘*Partnership working’*, where care homes were integrated within the multidisciplinary team as colleagues, strengthened provision of palliative and end-of-life care.

*then there became more palliative resources that came in*,* there were discussions between palliative and district nurses*,* the doctor started coming in*,* you know*,* the partnership working. I don’t know if the doctor came in*,* but we had more access to clinical competence.* [ID111 manager, large residential home]

Most care homes with on-site registered nurses sought palliative and end-of-life care support from specialist palliative care services (92% nursing and 88% dual, see additional file 3). Only 63% of residential care homes sought support from specialist palliative care services. Interviews highlighted that nursing homes used the palliative care teams to access specialist nursing, including tissue viability, hydration and frailty, to bridge the gap between care home nursing care and hospital care.

*we’ve got a palliative care network team who are happy to come in and help us with our wounds and our tissue viability. And that’s just one small area where we’ve now got far greater support. […]. Even if we are all nurses trying to look after people*,* sometimes there are things when you do need an expert to see you. […]. And it’s knowing that we’ve now got someone to support us.* [ID025 manager, medium nursing home]

In contrast, residential homes mostly relied on palliative and end-of-life care support from community nursing teams who provided clinical nursing care, including administering sub-cutaneous end-of-life medication. This worked particularly well when they worked in partnership with the GP practice and could provide staff with knowledge they otherwise lacked.

*We have a lot of support from the community matrons. It also works really well that we had a care coordinator from our doctor surgery*,* who could give us that little bit of extra knowledge when there was an end-of-life patient that we did have. We could work out the best way to make them feel comfortable. […] we’re not like a medical nursing home*,* we’re a residential home. There was a lot of things that we couldn’t do without district nurse input. So I think like administering end of life drugs and things like that. We have a lot of input from the community matrons which was so helpful to us.’ [*ID214 care worker, large residential home].

### Theme 3: ‘a shared language’ – use of guidance, tools and standardised care planning

The proactive use of end-of-life guidance, tools and care plans supported care homes to deliver care through a standardised way of working, which promoted confidence. All nursing home, 88% of dual and 63% of residential home respondents reported using guidance to deliver palliative and end-of-life care (additional file 3). Guidance varied from large, well-embedded initiatives, e.g. the Gold Standards Framework, to in-house guidance developed by experienced staff.

*I think from that point of view we were lucky*,* because we*,* you know*,* the [Gold Standards Framework]*,* we’ve been doing it since 2008*,* so by now it’s just firmly embedded*,* and nobody even gives it a second thought really. My nurses are really skilled at end-of-life care*,* anything that faces them*,* and those sort of difficult conversations aren’t that difficult anymore. [ID018 manager*,* large dual registration home]*

When care planning was undertaken using structured care plans completed with the resident, leaders could be confident that care was aligned to resident wishes in a standardised way. Involvement of external services and family ensured all were informed about the resident’s wishes for care in the future. Structured care planning supported staff knowledge of each resident which was particularly important for residential care home leaders to feel confident in the care they were providing.

*So we are quite a large residential home. When residents came in*,* especially during the COVID pandemic*,* we ensured that residents sat with their most trusted member of staff that they felt most comfortable with*,* and they designed an advance care plan. So they designed a plan where it highlighted sort of what they would want to happen during end-of-life*,* and what they would want us to prioritise. What funeral arrangements they would like*,* if they want us to tell the family anything. […] The ReSPECT* [*Recommended Summary Plan for Emergency Care and Treatment] forms denote how they want to be treated*,* whether they want to prioritise life or prioritise comfort. Whether they would want to remain at the home or go into hospital*. [ID214 care worker, large residential home]

Confidence that care was aligned to resident wishes was enhanced in small care homes as they were able to spend time with every resident to complete the documentation and have multiple conversations about wishes for care. Over 79% of small care homes reported using palliative and end-of-life care guidance (additional file 3).

*We’re only very*,* very small home*,* we’re a 22 bed home*,* it’s [name] that has the conversations and puts these things in place. So we had conversations with all of our residents. Even if they’ve got the ReSPECT* [*Recommended Summary Plan for Emergency Care and Treatment] forms in place to see if their priorities would be the same. [263 manager*,* small residential home]*

Standardised tools enabled non-clinical staff to better identify signs of nearness to end-of-life. Tools gave senior care leaders evidence of the resident’s condition and a shared language to communicate with external services. This included out of hours when external professionals have little knowledge of a resident’s condition and symptoms.

*So the use of the tool [a physical deterioration and escalation tool] was helpful in that*,* in that the soft signs became more evident and we were able to utilise that tool to out of hours people who don’t know -- that was helpful.* [ID026 deputy manager, large dual registration home]

## Discussion

Care home staff confidence to provide palliative and end-of-life care is influenced by care home registration type and size. Findings from the online survey indicated that nursing senior care leads were more confident compared to residential care senior care leads, irrespective of care home size. However, further exploration in qualitative interviews illuminated further understanding on the influence of registration type and size on confidence, constructed as three themes: feelings of preparedness, partnership working, and shared language.

‘Feelings of preparedness’ were embedded from clinical training of nursing staff. Training provided a foundation of confidence and preparedness that was consolidated and strengthened through mediating factors, such as care home infrastructure, support from external specialist services, and use of guidance. Nursing expertise also resulted in an implicit, peer level of trust from external services, fostering ‘partnership working’. Whereas staff from residential homes were more confident when they received empowering support from the GP and used established end-of-life care guidance and tools to provide them with a ‘shared language’ to communicate a resident’s needs or condition. However, findings are ambiguous as to why residential care homes accessed specialist palliative care services less than nursing homes. It may relate to the differences in complexity of care needs of nursing home residents compared to residential home residents.

The findings from interviews particularly highlighted how levels of confidence varied by care home size. Large care homes often benefitted from having embedded care structures and robust training avenues, enhanced by adequate staffing capacity. Conversely, small care homes required more clinical support to feel confident in decisions around care. However, a strength of small care homes was the intimate knowledge of residents and their wishes which boosted staff confidence in how they delivered care.

The participants in this study identified how care home staff benefit from training and support from external services, and the use of end-of-life tools and guidance. This aligns with existing literature promoting practical education [17, 25, 26], experience [17, 26, 27], mentorship [17], tools [28, 29] and guidance [30] to promote confidence. If care homes become the most common place to die and palliative care needs increase as predicted [31, 32], this need for training in palliative and end-of-life care for all staff will become an imperative. In England, the Enhanced Health in Care Homes framework already promotes recognition of expertise and collaboration with external services to deliver care [33]. However, this study goes further to advocate for collaboration and equitable structures to support delivery of palliative and end-of-life care that are adaptable to care home characteristics. This includes: a named GP practice(s) to provide a standard minimal level of primary care support (such as in-person visits for residential homes and direct, remote access for nursing homes); and access to training opportunities, including in-house training for small care homes.

In the USA, the ongoing UPLIFT-AD trial aims to implement structures to ensure more equitable access to palliative care across nursing homes [34]. Similarly, in Europe the PAlliative Care for older people in Europe (PACE) Steps to Success Programme aimed to implement a palliative care approach into nursing homes through staff training and standardised tools [35]. A process evaluation of the PACE programme found that encouraging staff to attend training required sufficient staffing resource and incentives (such as attendance certificates to aid career progression) [36]. However, current research primarily focuses on nursing homes. This study has identified how the mechanisms for enhancing staff confidence differ for residential care homes. Future research should focus on interventions to support residential care home staff confidence. In particular, optimal models of integration with specialist palliative care services for residential care homes to support palliative and end-of-life care.

### Strengths and weaknesses/limitations of the study

This study adds a nuanced exploration of the influence of care home registration type and size on staff confidence to provide palliative and end-of-life care. This nuance has been little explored in the literature, yet this understanding of context is vital to develop and implement appropriate interventions tailored to the care home setting. This tailoring could maximise the impact on staff confidence, and in turn, promote optimal palliative and end-of-life care for residents. The mixed methodology allowed for a novel, in-depth, qualitative explanation of confidence from a broad understanding provided by the quantitative data. While the use of preliminary quantitative survey data to develop the qualitative interview topic guide signalled important areas of interest, it may have also limited the scope of the interviews.

Further, we found a ceiling effect in the data, whereby many participants scored highly on the Palliative Care Self-Efficacy scale, with a median total score of 45 (of a maximum score of 48). Previous work has found that hospice nurses score highly on the scale [37] and that duration of nursing experience and resulting knowledge are predictors of self-efficacy in delivering palliative and end-of-life care [21]. As all participants in this study were in senior positions with high levels of experience, a high level of confidence might be expected. As a result, we took a data driven approach to analyse the quantitative data by dichotomising the sample based on median scores during analysis. Moreover, we did not collect data on membership to a care home chain, or CQC rating. Previous work found that CQC rating was correlated with staff confidence, with care homes rated as ‘outstanding’ scoring the highest [38]. We could not identify survey respondents who had dual roles within the care home, such as manager and nurse. This may have led to further insight into the effect of role on confidence.

Finally, it is important to note the timing of recruitment to the online survey. The survey allowed an opportunity to reflect on how end-of-life care is managed following a unique period of time, the COVID-19 pandemic. Care homes were extremely overstretched which may have led to a reduced capacity and lower priority to take part in research, particularly for small care homes with limited staff resource.

## Conclusions

Care home registration type and size influences staff confidence to deliver palliative and end-of-life care. Overall, nursing home senior care leaders are more confident compared with leaders from residential care homes. Confidence was promoted through feelings of preparedness, partnership working with multi-disciplinary external services and a shared language developed from end-of-life guidance. This study has found distinct differences in how confidence is promoted by care home registration type. Future work should focus on effective mechanisms for improving confidence in residential care homes and how palliative care services might be better integrated with residential care homes.

### Electronic supplementary material

Below is the link to the electronic supplementary material.

Supplementary Material 1

Supplementary Material 2

Supplementary Material 3

## Data Availability

Applications for sharing of the survey data can be made and will be considered on a case-by-case basis on receipt of a methodological sound proposal to achieve aims in line with the original protocol. The study protocol is available on request. All requests for data access should be addressed to the Chief Investigators, Professors Katherine Sleeman and Catherine Evans via the details on the CovPall Care Homes website (https://www.kcl.ac.uk/research/covpall-care-homes and palliativecare@kcl.ac.uk) and will be reviewed by the Study Steering Group.

## Abbreviations

CI: Confidence Interval
CQC: Care Quality Commission
GP: General Practitioner
IQR: Interquartile Range
OR: Odds Ratio
