# Supplementary material for: The influence of care home registration type and size on senior care leader’s confidence to provide palliative and end-of-life care: an explanatory sequential mixed methods study
Source: BMC Palliat Care. 2024 Aug 22;23:213. doi: 10.1186/s12904-024-01525-0 (PMC11340158; doi:10.1186/s12904-024-01525-0)
Supplement: Supplementary file 1 — Supplementary Material 1 [file 12904_2024_1525_MOESM1_ESM.docx]

# Additional file 1

## Good Reporting of A Mixed Methods Study (GRAMMS)

| (1) Describe the justification for using a mixed methods approach to the research question | Page 4 |
| --- | --- |
| (2) Describe the design in terms of the purpose, priority and sequence of methods | Page 4-7 |
| (3) Describe each method in terms of sampling, data collection and analysis | Pages 4-7 |
| (4) Describe where integration has occurred, how it has occurred and who has participated in it | Page 7 |
| (5) Describe any limitation of one method associated with the present of the other method | Page 19 |
| (6) Describe any insights gained from mixing or integrating methods | Page 17-20 |
