# Supplementary material for: The influence of care home registration type and size on senior care leader’s confidence to provide palliative and end-of-life care: an explanatory sequential mixed methods study
Source: BMC Palliat Care. 2024 Aug 22;23:213. doi: 10.1186/s12904-024-01525-0 (PMC11340158; doi:10.1186/s12904-024-01525-0)
Supplement: Supplementary file 2 — Supplementary Material 2 [file 12904_2024_1525_MOESM2_ESM.docx]

# Additional file 2

## Palliative Care Self-Efficacy Scale

**Please rate your degree of confidence with the following patient / family interactions and patient management topics, by ticking the relevant box below**

| 1 = Need further basic instruction | | 2 = Confident to perform with close supervision / coaching | | | | |
| --- | --- | --- | --- | --- | --- | --- |
| 3 = Confident to perform with minimal consultation | | 4 = Confident to perform independently | | | | |
| No | Patient/family interactions and clinical management | | 1 | 2 | 3 | 4 |
| 1 | Answering patients questions about the dying process | |  |  |  |  |
| 2 | Supporting the patient or family member when they become upset | |  |  |  |  |
| 3 | Informing people of the support services available | |  |  |  |  |
| 4 | Discussing different environmental options (eg hospital, home, family) | |  |  |  |  |
| 5 | Discussing patient’s wishes for after their death | |  |  |  |  |
| 6 | Answering queries about the effects of certain medications | |  |  |  |  |
| 7 | Reacting to reports of pain from the patient | |  |  |  |  |
| 8 | Reacting to and coping with terminal delirium | |  |  |  |  |
| 9 | Reacting to and coping with terminal dyspnoea (breathlessness) | |  |  |  |  |
| 10 | Reacting to and coping with nausea / vomiting | |  |  |  |  |
| 11 | Reacting to and coping with reports of constipation | |  |  |  |  |
| 12 | Reacting to and coping with limited patient decision-making capacity | |  |  |  |  |
