# Supplementary material for: The influence of care home registration type and size on senior care leader’s confidence to provide palliative and end-of-life care: an explanatory sequential mixed methods study
Source: BMC Palliat Care. 2024 Aug 22;23:213. doi: 10.1186/s12904-024-01525-0 (PMC11340158; doi:10.1186/s12904-024-01525-0)
Supplement: Supplementary file 3 — Supplementary Material 3 [file 12904_2024_1525_MOESM3_ESM.docx]

# Additional file 3

Table A. Description of external services accessed, and guidance used by care home type and size

|  | | **Residential home (n=49)** | | | **Nursing home (n=24)** | | | **Mixed registration home (n=34)** | | |
| --- | --- | --- | --- | --- | --- | --- | --- | --- | --- | --- |
|  |  | **Small (n=25)** | **Medium**  **(n=18)** | **Large (n=6)** | **Small**  **(n=3)** | **Medium**  **(n=12)** | **Large (n=9)** | **Small (n=1)** | **Medium (n=10)** | **Large (n=23)** |
| If you need advice about palliative and end of life care, who do you usually ask? Responded yes | GP | 18  (72) | 16  (89) | 2  (33) | 2  (67) | 10  (83) | 7  (78) | 1  (100) | 9  (90) | 17  (74) |
|  | Community nurses | 19  (76) | 15  (83) | 2  (33) | 0  (0) | 1  (8) | 2  (22) | 0  (0) | 1  (10) | 7  (30) |
|  | Specialist palliative care service or hospice | 12  (48) | 13  (72) | 6  (100) | 3  (100) | 11  (92) | 8  (89) | 1  (100) | 10  (100) | 19  (83) |
|  | Community pharmacist | 8  (32) | 5  (27) | 1  (17) | 1  (33) | 1  (8) | 2  (22) | 0  (0) | 1  (10) | 3  (13) |
|  | Other community services | 6  (24) | 2  (11) | 0  (0) | 0  (0) | 1  (8) | 1  (11) | 0  (0) | 2  (20) | 1  (4) |
|  | Geriatrician | 2  (8) | 0  (0) | 0  (0) | 0  (0) | 1  (8) | 0  (0) | 0  (0) | 1  (10) | 2  (9) |
|  | Other | 1  (4) | 2  (11) | 0  (0) | 0  (0) | 0  (0) | 0  (0) | 0  (0) | 1  (10) | 0  (0) |
| Palliative and end of life care guidance used | | 19  (76) | 13  (72) | 0  (0) | 3  (100) | 12  (100) | 9  (100) | 1  (100) | 10  (100) | 19  (83) |
